# SELF-REPORTED VISION IMPAIRMENT AND SUBJECTIVE WELL-BEING IN OLDER ADULTS: A LONGITUDINAL MEDIATION ANALYSIS

**DOI:** 10.1093/geroni/igz038.2033

**Published:** 2019-11-08

**Authors:** Joshua R Ehrlich, Xiaoling Xiang, Khushali Shah, Rita X Hu, Brian C Stagg, Vicki A Freedman

**Affiliations:** 1 University of Michigan, Ann Arbor, Michigan, United States; 2 University of Miami, Miami, Florida, United States; 3 University of Michigan, Ann Arbor, Ann Arbor, Michigan, United States; 4 Duke University, Durham, North Carolina, United States

## Abstract

Vision impairment (VI) in older adults is associated with declines in well-being. However, the pathways through which poor vision leads to declines in well-being have not been well described. The purpose of this study was to determine whether activity limitations and social participation restrictions mediate the impact of self-reported VI on subjective well-being. This study used data from the National Health and Aging Trends Study (NHATS), a nationally-representative longitudinal study of Medicare beneficiaries 65 and older that includes detailed measures of the disablement process. We conceptualized a longitudinal mediation model linking self-reported VI and subjective well-being. Structural equation modeling was used to test the mediating effects of activity limitations and social participation restrictions while adjusting for covariates. The final sample included 5,431 respondents. At baseline, 8.0% of Medicare beneficiaries had self-reported VI. Subjective well-being scores were significantly lower among respondents with self-reported VI (15.7, 95% CI=15.2, 16.2) compared to those without VI (17.6, 95% CI=17.5, 17.7). Self-reported VI had a significant indirect effect on subjective well-being through limiting mobility (β=-.04, 95% CI=-.07, -.03) and household activities (β=-.05, 95% CI=-.08, -.03), but not self-care limitations (β=0.0, 95% CI=0.0, 0.0) or participation restrictions (β=0.0, 95% CI=-0.01, 0.00). Total indirect effects from all mediation paths accounted for 42% of the effect of VI on well-being. In conclusion, mobility and household activity limitations are significant mediators that explain a considerable portion of the impact of poor vision on well-being. Interventions to promote successful accommodation may result in greater well-being for visually impaired older adults.

